# Application of recombinant TAF3 PHD domain instead of anti-H3K4me3 antibody

**DOI:** 10.1186/s13072-016-0061-9

**Published:** 2016-03-22

**Authors:** Goran Kungulovski, Rebekka Mauser, Richard Reinhardt, Albert Jeltsch

**Affiliations:** Faculty of Chemistry, Institute of Biochemistry, University Stuttgart, Pfaffenwaldring 55, 70569 Stuttgart, Germany; Max-Planck-Genomzentrum Köln, Carl-von-Linné-Weg 10, 50829 Cologne, Germany

**Keywords:** Histone H3K4 methylation, Reading domains, Epigenetics, Chromatin, Antibody

## Abstract

**Background:**

Histone posttranslational modifications (PTMs) represent a focal point of chromatin regulation. The genome-wide and locus-specific distribution and the presence of distinct histone PTMs is most commonly examined with the application of histone PTM-specific antibodies. In spite of their central role in chromatin research, polyclonal antibodies suffer from disadvantages like batch-to-batch variability and insufficient documentation of their quality and specificity.

**Results:**

To mitigate some of the pitfalls of using polyclonal antibodies against H3K4me3, we successfully validated the application of a recombinant TAF3 PHD domain as anti-H3K4me3 affinity reagent in peptide array, western blot and ChIP-like experiments coupled with qPCR and deep sequencing.

**Conclusions:**

The successful addition of the TAF3 PHD domain to the growing catalog of recombinant affinity reagents for histone PTMs could help to improve the reproducibility, interpretation and cross-laboratory validation of chromatin data.

**Electronic supplementary material:**

The online version of this article (doi:10.1186/s13072-016-0061-9) contains supplementary material, which is available to authorized users.

## Background

The N-terminal tails of histones stick out from the core nucleosome and are subject to more than a 100 of posttranslational modifications (PTMs) [1, 2]. Many of these histone PTMs lie in the focal point of chromatin regulation and contribute to numerous cellular processes such as development and disease [3–5]. Due to their central role in essential cellular processes, their systematic mapping has been a prime concern of many scientific consortia such as ENCODE, Roadmap Epigenomics, Blueprint Epigenome and IHEC. The genome-wide and locus-specific density and distribution of distinct histone PTMs is typically examined with the help of histone PTM-specific antibodies, which are used to precipitate modified chromatin, followed by further downstream analyses. Because of this, the antibody is the principal mediator relaying the information about the presence and distribution of an individual histone modification, and its properties should be of highest quality and well documented [6]. However, there are several constitutional difficulties of recognizing histone PTM epitopes by antibodies, because of their small size, the chemical similarity of numerous histone PTMs, the hypermodified nature of histone tails where one modification can influence the detection of another, and the similarities in the amino acid motifs surrounding the modified residues. Hence, critical properties of antibodies include various features such as their specificity with respect to the tested modification and its amino acid sequence context, their cross-reactivity and their response to the presence of secondary modifications. This inherent complexity is further exacerbated by the large variability of these critical properties between antibody lots (in case of polyclonal antibodies). Moreover, even broadly used antibodies occasionally lack the expected specificity, and the quality of datasheets provided by companies is insufficient in general [6–12].

To mitigate some of the above-mentioned drawbacks related to histone PTM antibodies, recently we have devised a strategy of using recombinant histone modification interacting domains (HiMIDs) instead of antibodies in different experimental settings such as western blot and chromatin precipitation coupled with deep sequencing [9]. This approach is complementary to alternative strategies of using recombinant antibodies in ChIP experiments [13] as well as using recombinant HiMIDs in chromatin precipitation studies coupled with mass spectrometry [14]. The cheap production of recombinant HiMIDs in *Escherichia coli*, their amenability to protein engineering and the unlimited availability of recombinant proteins with constant properties have the potential to solve many of the contemporary problems in chromatin biology such as lab-to-lab and long-term reproducibility of results, and insufficient documentation of the intricate critical properties of affinity reagents [6, 11].

So far we have validated recombinant HiMIDs against histone PTMs such as H3K9me3, H3K9me3/K4unmodified, H3K27me3 and H3K36me3 [9]. However, no HiMID was available to study the lysine 4 trimethylation of histone H3 (H3K4me3), which is one of the most important and widely studied histone marks, highly conserved from yeast to mammals [15, 16]. It is considered a landmark of promoters, especially promoters of active genes [17]. The TATA box-binding protein-associated factor 3 (TAF3) protein is involved in anchoring the TFIID basal transcription factor to nucleosomes containing H3K4me3 through its PHD domain, thereby stimulating pre-initiation complex formation [18, 19]. Moreover, the interaction of TAF3 PHD and H3K4me3 is involved in p53-dependent regulation of genes upon genotoxic insults [19]. In this study, we systematically examined the applicative potential of the TAF3 PHD domain [18, 20] in chromatin biology as a substitute of anti-H3K4me3 antibodies. Herein, we demonstrate that the TAF3 PHD domain performs similarly to ENCODE-validated antibodies in western blot and ChIP-seq-like experiments highlighting its applicative potential to replace anti-H3K4me3 antibodies.

## Results

Previous studies have demonstrated that the recombinant TAF3 PHD domain can be used to precipitate H3K4me3-enriched chromatin [18]. In order to investigate the applicative capabilities of the recombinant GST-tagged TAF3 PHD domain as an H3K4me3 affinity reagent, we closely followed the quality control guidelines set by the ENCODE consortium [7, 21] and recently upgraded by us [6]. The set of guidelines dictates that the tested affinity reagent (antibody or reading domain) should perform successfully in at least three out of four key experiments: (1) specific binding to peptide arrays or another high-throughput peptide-based platform; (2) binding to native histones and lack of binding to recombinant histones in western blot experiments; (3) loss of signal in western blot experiments with native histones, where the targeted histone PTM has been depleted; and (4) successful precipitation of chromatin, reproducibility of ChIP-seq data and high correlation with validated ChIP-seq datasets.

### Peptide array and western blot analyses of TAF3 PHD and anti-H3K4me3 antibody

**Fig. 1:**
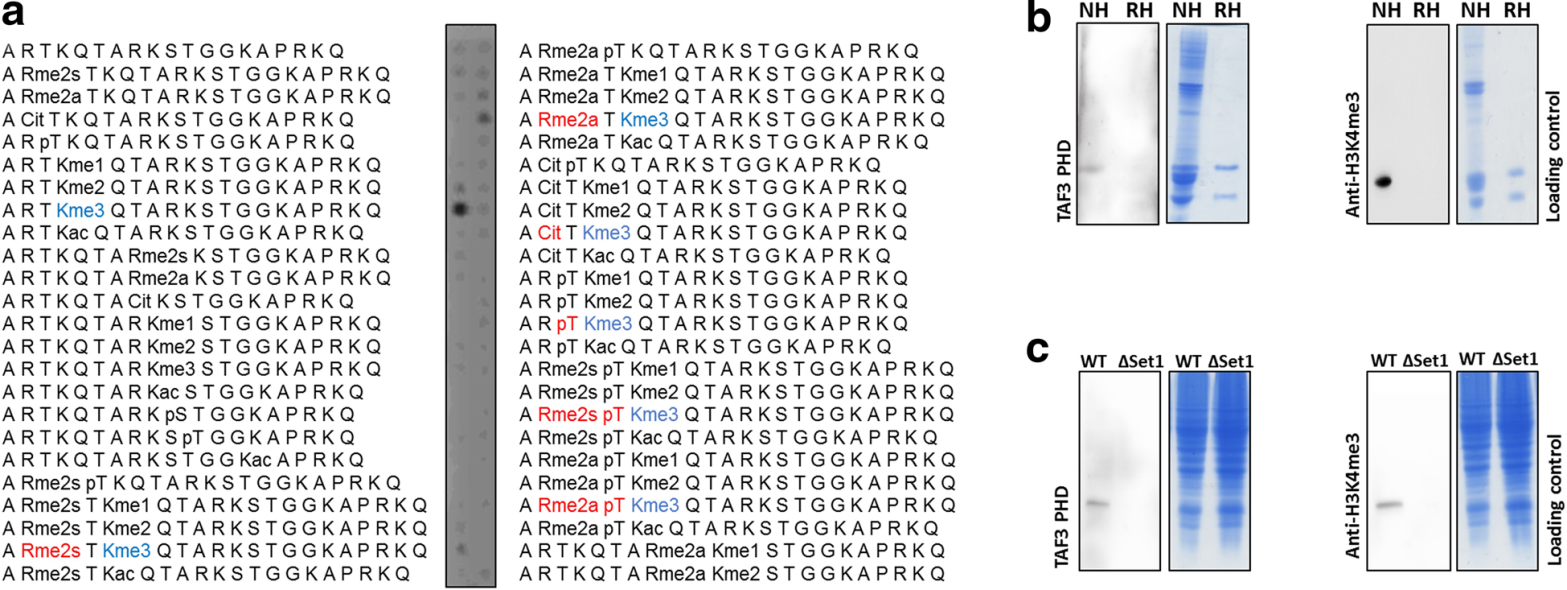
Specificity analyses of TAF3 PHD and anti-H3K4me3 antibody. **a** Peptide array profiling of TAF3 PHD. **b** Western blot analyses with TAF3 PHD and anti-H3K4me3 antibody with native histones carrying H3K4me3 (NH) and recombinant histones (RH) which are free of histone PTMs. **c** Western blot analyses with TAF3 PHD and anti-H3K4me3 antibody with lysates isolated from *S. cerevisiae* wild-type (WT) or Set1 knockout (ΔSet1) cells, which do not contain H3K4me3

First, we probed the TAF3 PHD domain on CelluSpots peptide arrays, which have been used in previous reports to study the specificity of histone PTM reading domains [22–25]. The TAF3 PHD domain exhibited binding to H3K4me3-modified peptides. Focusing on the effects of adjacent marks, we observed inhibition of binding in the presence of H3R2me2 (as reported before [18, 20]), H3T3ph (as reported before [26]) and H3R2citr (Fig. 1a). We did not observe a trimethyllysine-specific binding at any other lysine residues (data not shown). The H3K4me3-specific interaction was confirmed with a TAF3 PHD M882A mutant that has been reported to have a diminished binding affinity to H3K4me3 peptides [18, 20]. The recombinant TAF3 PHD M882A mutant was generated and purified with similar yield and purity as the TAF3 PHD WT (Additional file 1: Fig. S1A) but did not show interaction on peptide arrays (Additional file 1: Fig. S1B).

To additionally confirm the PTM-dependent interaction and also check for possible cross-reactivity with modified non-histone proteins, we carried out far-western blot experiments with nuclear extracts containing native histones and recombinant histones H3 and H4, where the TAF3 PHD reading domain was used instead of an anti-H3K4me3 antibody. The domain bound specifically to native histones, but not recombinant histones, which do not carry any PTMs (Fig. 1b). The dependence of the interaction on an intact H3K4me3 binding pocket was again validated with a TAF3 PHD M882A pocket mutant (Additional file 1: Fig. S1C). To further verify that the interaction was dependent on the presence of the H3K4 methylation mark, we isolated histones from *Saccharomyces cerevisiae* wild-type (WT) and Set1 knockout cells (ΔSet1) and carried out western blot experiments. Set1 is the only H3K4 methyltransferase in *S. cerevisiae*, so that histones isolated from Set1 knockout cells are free of H3K4me3. In agreement with the previous data, the TAF3 PHD domain showed specific binding to histones isolated from WT cells, but not to histones isolated from Set1 KO cells (Fig. 1c), again confirming the specificity of interaction.

### Chromatin precipitation coupled with deep sequencing of TAF3 PHD and anti-H3K4me3 antibody

**Fig. 2:**
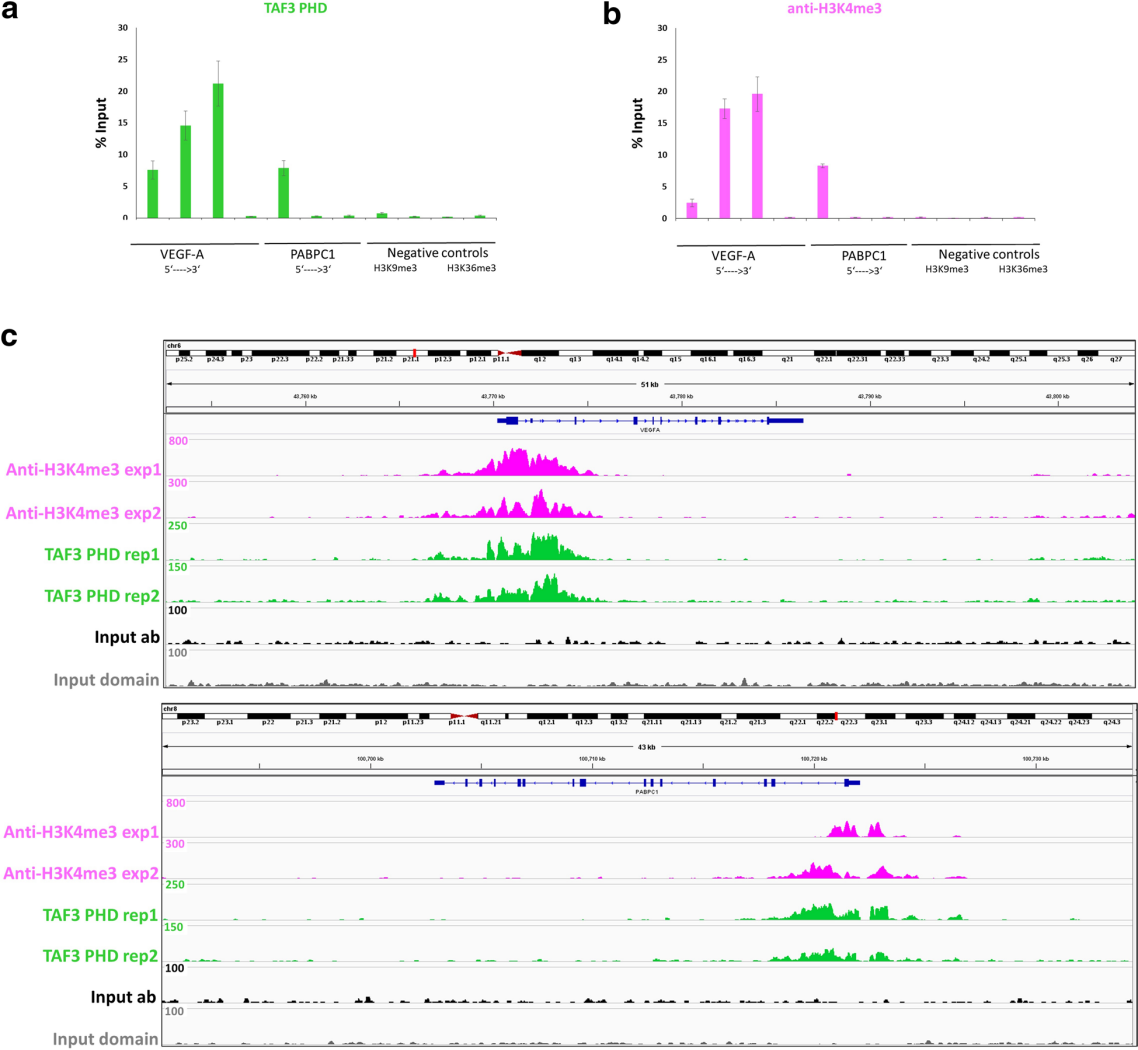
CIDOP and ChIP carried out with TAF3 PHD and anti-H3K4me3 antibody. **a** CIDOP-qPCR signals using amplicons covering the *VEGF*-*A*, *PABPC1* loci and control regions. For details about the positions of the *VEGF*-*A* and *PABPC1* amplicons, refer to (Additional file 1: Fig. S2A, B). **b** ChIP-qPCR signals using the same amplicons as **a**. **c** Representative genome browser views comparing CIDOP-seq and ChIP-seq (from ENCODE) signals taken from both experiments/replicates for each method, respectively. Zoom in at the *VEGF*-*A* locus (*upper panel*) and *PABPC1* locus (*lower panel*). Note the difference in the *PABPC1* locus between anti-H3K4me3 experiment 1 and all three other datasets. For additional browser views, refer to Additional file 1: Fig. S5A

Chromatin immunoprecipitation (ChIP) is by far the most prevalent method for studying the genomic presence and distribution of histone PTMs. For this aim, we sought to evaluate the performance of the TAF3 PHD domain in ChIP-like experiments, which we call chromatin interacting domain precipitation (CIDOP). The chromatin used for our studies consisted primarily of mononucleosomes (Additional file 1: Fig. S2C) isolated from HepG2 cells, and ChIP experiments with a widely used anti-H3K4me3 antibody were performed in parallel with the CIDOP experiments. First, we validated the specificity of the precipitation with quantitative PCR of amplicons covering promoter-proximal and promoter-distal regions of the *VEGF*-*A* and *PABPC1* genes, and additional H3K36me3- and H3K9me3-enriched regions (Fig. 2a, b; Additional file 1: Fig. S2A, B). Both, the TAF3 PHD domain and the anti-H3K4me3 antibody, performed similarly by specifically enriching for nucleosomes in promoter-proximal regions and showing residual binding for promoter-distal and control regions. The specificity was further validated with the TAF3 PHD M882A pocket mutant, where only non-specific residual binding was observed (Additional file 1: Fig. S3).

**Fig. 3:**
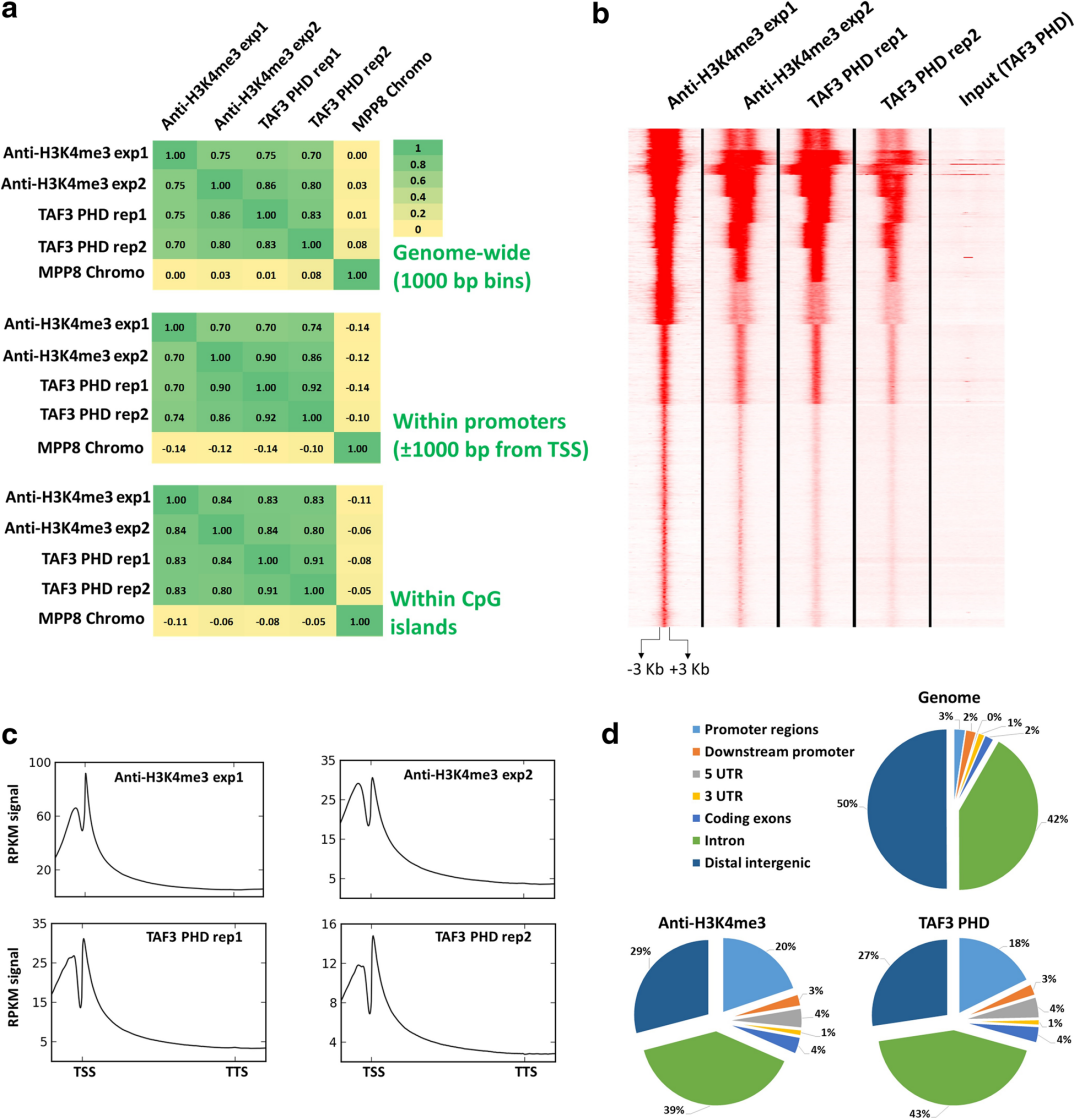
CIDOP-seq and ChIP-seq carried out with TAF3 PHD and anti-H3K4me3 antibody. **a** Spearman’s correlation coefficient in 1-kb bins genome-wide (*upper heatmap*) or Pearson’s correlation coefficient between CIDOP-seq and ChIP-seq signals within promoters (*middle heatmap*) and within CpG islands (*lower heatmap*). **b** Clustering analysis of tag densities from TAF3 PHD and anti-H3K4me3 datasets. Tags were collected in 6-kb windows, centered on the midpoints of anti-H3K4me3 exp1 peaks (which was the experiment with the highest number of detected peaks) and sorted by *k*-means clustering (ten clusters). **c** Composite profiles of TAF3 PHD and anti-H3K4me3 antibody distribution over metagenes fitted in 5-kb gene bodies and 1-kb flanks from the TSS/TTS. **d** Distribution of CIDOP-seq and ChIP-seq peaks among different genomic elements

Our next objective was to extend the CIDOP-qPCR experiments to a genome-wide level. We carried out CIDOP-seq experiments, where our data were compared head to head with two ChIP-seq datasets obtained with anti-H3K4me3 antibodies in HepG2 cells available from ENCODE. Our CIDOP-seq data showed high concordance with both ChIP-seq datasets at the *VEGF*-*A* locus, but some differences were observed with the antibody dataset 1 (but not dataset 2) at the *PABPC1* locus (Fig. 2c), again highlighting the possible discrepancies emerging from using two different antibodies against the same histone PTM in the ENCODE data. Nevertheless, detailed genome-wide analyses demonstrated a high concordance between our CIDOP-seq data and both ENCODE ChIP-seq datasets (Fig. 3; Additional file 1: Fig. S5A).

### Genomic distribution of TAF3 CIDOP-seq signal

We next tested the genome-wide distribution of the TAF3 CIDOP signal and its enrichment in particular genomic elements and compared the results with corresponding analyses of the H3K4me3 ChIP data. First, the TAF3 PHD CIDOP-seq and anti-H3K4me3 ChIP-seq datasets showed high genome-wide correlation of raw read densities with each other but not with H3K9me3 signal obtained with the MPP8 chromo domain used as an outgroup here (Fig. 3a). The H3K4me3 modification has been reported to be enriched in promoters and CpG islands [17, 27] where we observed a similarly high correlation of signals (Fig. 3a). Peak calling was carried out for all datasets, and the highest number of peaks was detected in antibody experiment 1. We performed k-means clustering of our CIDOP-seq together with the ENCODE H3K4me3 ChIP-seq raw read densities centered around the peaks identified in antibody experiment 1 (Fig. 3b). We observed clear signal at all peaks in all experiments illustrating the high concordance of all datasets. However, the signal intensity differed at some clusters, which caused variances in the number of called peaks in individual experiments. Nevertheless, the distributions of peaks obtained with both methods showed high overlap with each other, and between their respective replicates as well (Additional file 1: Fig. S4). Most importantly, the technical variances in peak distributions between experiments and repeats were of similar magnitude as the variances between CIDOP and ChIP. Next, we examined and compared the distribution of CIDOP-seq and ChIP-seq signals in a metagene analysis of all human genes and observed very similar signal distributions, peaking around the TSS and decreasing inside the gene body (Fig. 3c). Moreover, both datasets showed almost identical peak distributions in genomic elements, with high enrichment in promoters as expected (Fig. 3d). Next, we performed k-means clustering of our CIDOP-seq together with the ENCODE H3K4me3 and RNA pol II ChIP-seq datasets centered on CpG islands and promoters. We observed enrichment of H3K4me3 signal (detected by domain and antibodies) in RNA pol II rich clusters, again confirming the legitimate distribution of our data (Additional file 1: Fig. S5B, C).

**Fig. 4:**
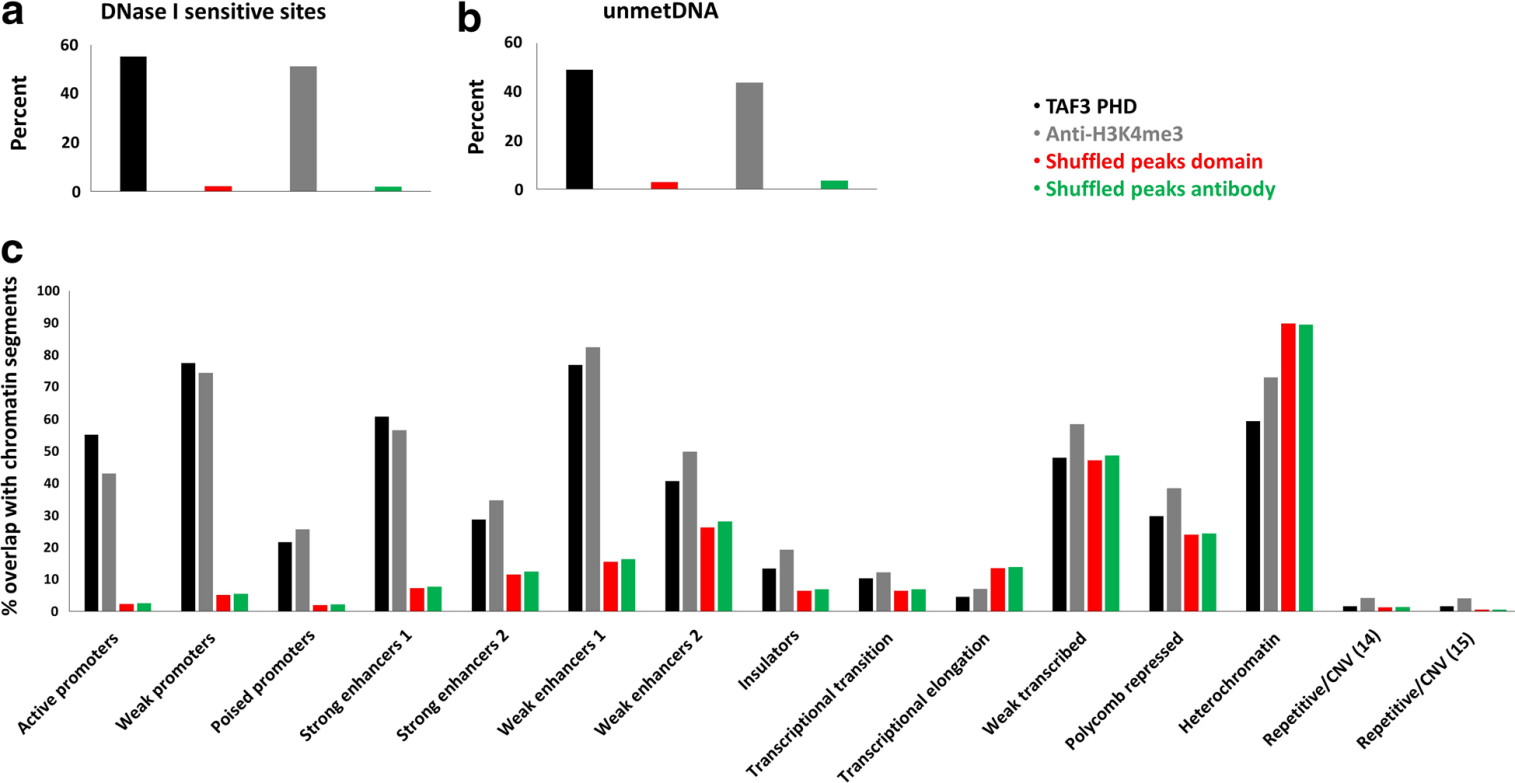
CIDOP-seq and ChIP-seq carried out with TAF3 PHD and anti-H3K4me3 antibody. **a** Percent of at least 10 % overlap of TAF3 PHD and anti-H3K4me3 peaks (merged from both replicates or experiments, respectively) and randomized shuffled genomic coordinates of the same number, with DNase I-sensitive sites. **b** Same analysis carried out with unmethylated DNA from RRBS data. **c** Overlap of H3K4me3 signals with different chromatin segments defined by Ernst et al. [30]. Please note the enrichment in segments associated with actively transcribed chromatin

Since the H3K4me3 mark is typically found within open chromatin and unmethylated CpG islands [28, 29], we examined its overlap with DNase I-hypersensitive sites and RRBS data of unmethylated CpGs, from ENCODE. Peaks obtained from TAF3 PHD CIDOP-seq and anti-H3K4me3 ChIP-seq showed similar overlap with open chromatin and unmethylated CpGs (Fig. 4a, b). Lastly, we explored the distribution of peaks from both datasets in different chromatin segments and repeats as defined by Ernst et al. [30]. The enrichment in actively transcribed chromatin segments and depletion in repeat elements further demonstrated the similarity between the CIDOP-seq and ChIP-seq datasets and their proper genomic enrichment and distribution (Fig. 4c; Additional file 1: Fig. S6).

## Discussion

Antibodies have been “the jack of all trades” in biology in the past century and remain in the focal point of experimental chromatin biology, especially in translating the complex syntax of the language of histone marks into properties that can be experimentally analyzed. H3K4me3 is one of the best characterized histone PTMs, although its direct and multifaceted functional role in transcription is still not fully understood [15, 16, 31]. The importance of this histone mark in basic chromatin biology makes the development of novel H3K4me3-specific affinity tools and the refinement of old ones a principal goal in molecular biotechnology. We screened several potential H3K4me3 readers such as BPTF-PHD [32], RAG2-PHD [33] and found TAF3-PHD [18] the most promising in our hands. Another study recently showed that the ING2-PHD might work for this type of applications as well [14].

In this work, we demonstrated that the recombinant TAF3 PHD domain can be used as a specific, reliable and reproducible alternative to anti-H3K4me3 antibodies. With rigorous characterization of its experimental performance, following established guidelines and criteria [6, 7, 11, 21], we have included another member to the growing catalog of recombinant affinity reagents for histone PTMs [9, 13]. These reagents are characterized by a consistent performance and the absence of lot-to-lot variability due to recombinant production. The potential problems caused by lot-to-lot variance in antibody properties are illustrated in our data, where the two H3K4me3 datasets available from ENCODE showed a larger variation than the two repeats of our TAF3 PHD-based CIDOP experiments. While other reasons cannot be excluded, one critical parameter in the ENCODE H3K4me3 ChIP data is the application of two different antibodies. Unfortunately, this question cannot be fully resolved, because the corresponding antibody batches are no longer available, so that no retrospective specificity analyses of the antibodies and ChIP-seq can be conducted. In contrast, due to their recombinant production, the availability of recombinant reading domains is unlimited and future batches will show identical properties (if the purification procedures are not altered), allowing for unlimited follow-up studies and experimental reproductions.

## Conclusions

Trimethylation of histone H3 at K4 is an important histone PTM associated with active promoters. The work presented here, including western blot, CIDOP-qPCR and CIDOP-seq, clearly illustrates that the TAF3 PHD domain can be applied as a reliable substitute of anti-H3K4me3 antibodies. The continuous growth of the catalog of recombinant reagents with constant and well-documented properties should foster the replacement of polyclonal antibodies by HiMIDs in routine applications. This could help to improve the reproducibility, interpretation and cross-laboratory validation of chromatin data.

## Methods

### Cloning, site-directed mutagenesis, expression and purification

The sequence encoding the PHD domain from TAF3 (amino acids 856–929 of NCBI Reference Sequence NP_114129.1.1) was amplified from cDNA and cloned as a GST fusion protein into pGEX-6p-2 vector (GE Healthcare). It was overexpressed at 20 °C in the presence of 50 µM zinc in LB medium, induced 1 mM IPTG at 0.6–0.8 OD_600_ and purified by affinity chromatography as described [34]. The M882A mutation was introduced by site-directed mutagenesis [35] and validated by restriction analysis and Sanger sequencing.

### Peptide arrays and western blot analyses

For western blot, native histones were isolated by acid extraction [36] from HEK293 cells and recombinant histones H3 and H4 were purchased from New England Biolabs. Two and a half micrograms of native histones and one microgram of recombinant histone H3 and H4 each were electrophoresed on a 16–18 % SDS-PA gel and transferred on a nitrocellulose membrane. Lysates from *S. cerevisiae* were isolated by bead beating, precipitated with 0.2 M H_2_SO_4_ and boiled in LAP. From now on, the CelluSpots peptide arrays (Active Motif, Carlsbad, CA, USA) and nitrocellulose membranes were treated the same.

The CelluSpots peptide arrays or nitrocellulose membranes were blocked by incubation in TTBS (10 mM Tris/HCl pH 7.5, 0.05 % Tween-20 and 150 mM NaCl) containing 5 % skim milk at +4 °C overnight, then washed two times with TTBS and one time with interaction buffer (300 mM KCl, 20 mM HEPES pH 7.5, 0.1 mM DTT and 10 % glycerol) and incubated with 10 nM of recombinant TAF3 PHD in interaction buffer for 2 h. Afterward, the arrays or membranes were washed two times with interaction buffer (with 300–500 mM KCl) and once with TTBS and incubated with primary anti-GST antibody for 1 h. After three washes with TTBS, the arrays or membranes were incubated with secondary anti-goat antibody for 1 h. Details regarding the protocol and the bioinformatic analysis are described in [37]. The antibodies were incubated with the membranes following the manufacturer’s recommendations for western blot.

### Chromatin precipitation and deep sequencing analysis

Native nucleosomes were isolated from HepG2 cells by micrococcal digestion of intact nuclei obtained as described [38]. Upon isolation of nuclei and micrococcal nuclease digestion, the protocol was modified in the following way: The MNase digestion was stopped with 2 mM EGTA and afterward the sample was sonicated for five cycles (20 s pulse, 30 s pause) with EpiShear Sonicator (Active Motif, Carlsbad, CA, USA). After sonication, the sample was centrifuged at 13,000×*g* for 10 min, and the resulting supernatant was taken as a nucleosomal fraction (predominantly nucleosomes). Then, native chromatin (30–60 µg, based on DNA absorbance) was incubated with 30–60 µg of recombinant TAF3 PHD, or anti-H3K4me3 antibody (ab8580, lot: GR85670-1), used following the manufacturer’s recommendations) in DP buffer (16.7 mM Tris–Cl, 167 mM NaCl, 1.1 % Triton X-100 and protease inhibitors) overnight with rotation. The domain–chromatin complexes were immobilized on 20–40 µl glutathione Sepharose 4B beads (GE Healthcare), washed three times with PB300 buffer (50 mM Tris–Cl, 300 mM NaCl, 0.5 % Nonidet P-40, 2 mM DTT). Elution, DNA recovery and qPCR were done essentially as described [9]. The primer sequences used in this study are shown in Additional file 1: Table S1.

Before proceeding to Illumina sequencing on the HiSeq 2500 platform, the quality of DNA precipitation and library generation was checked with Bioanalyzer (Agilent Technologies, Santa Clara, CA, USA). Around 10–15 million 100-nt sequence reads were obtained and mapped to hg38 with bowtie2, using the default settings [39] within the Galaxy Platform [40], retaining only uniquely mapped reads. Peaks were called with MACS [41] in Galaxy using the default settings, except in the case of the TAF3 PHD replicate 2, where *m*-fold of 20 instead of 32 was used. The peak annotation was done with CEAS within the Cistrome platform [42, 43], and the peak distribution and overlap with repeats and chromatin segments was determined with EpiExplorer [44]. The seqMINER tool was used for k-means clustering and heatmap generation [7], while the metagene profiles were generated in DeepTools [45].

The Spearman correlation of raw data in 1-kb bins was done in DeepTools as well, but the Pearson correlation within CpG islands and promoters was carried out in SeqMonk (http://www.bioinformatics.babraham.ac.uk/projects/seqmonk/) in window bins of 100 bp. Raw density profiles were generated in BEDTools [46], while RPKM normalized density profiles were generated in DeepTools [45] and visualized in the Integrative Genomics Viewer [47]. The raw sequencing data for all ChIP-seq (H3K4me3) experiments were downloaded from ENCODE, data from Broad Institute: (http://hgdownload.cse.ucsc.edu/goldenPath/hg19/encodeDCC/wgEncodeBroadHistone/). The RNA pol II ChIP-seq data were downloaded from ENCODE, data from Myers lab: (wgEncodeHaibTfbsHepg2Pol2Pcr2xRawDataRep2.fastq.gz).

### Data access

The CIDOP-seq raw data have been submitted to the ArrayExpress database (https://www.ebi.ac.uk/arrayexpress/) under the accession number E-MTAB-4103. The raw input and H3K9me3 CIDOP-seq data were taken from [9] and can be found under the accession number E-MTAB-2143.

## Additional file

10.1186/s13072-016-0061-9 Supplementary figures and table.

## Abbreviations

ChIP: chromatin immunoprecipitation
CIDOP: chromatin interacting domain precipitation
HiMID: histone modification interaction domain
PHD: plant homeodomain
PTM: posttranslational modification
TAF3: TATA box-binding protein (TBP)-associated factor 3
qPCR: quantitative PCR
