# Supplementary material for: Application of recombinant TAF3 PHD domain instead of anti-H3K4me3 antibody
Source: Epigenetics Chromatin. 2016 Mar 22;9:11. doi: 10.1186/s13072-016-0061-9 (PMC4802638; doi:10.1186/s13072-016-0061-9)
Supplement: Supplementary file 1 — 10.1186/s13072-016-0061-9 Supplementary figures and table. [file 13072_2016_61_MOESM1_ESM.pdf]

Application of recombinant TAF3 PHD domain instead of anti-H3K4me3 antibody

Goran Kungulovski, Rebekka Mauser, Richard Reinhardt and Albert Jeltsch

Supplementary figures S1-S6

Supplementary table S1

Supplementary figures

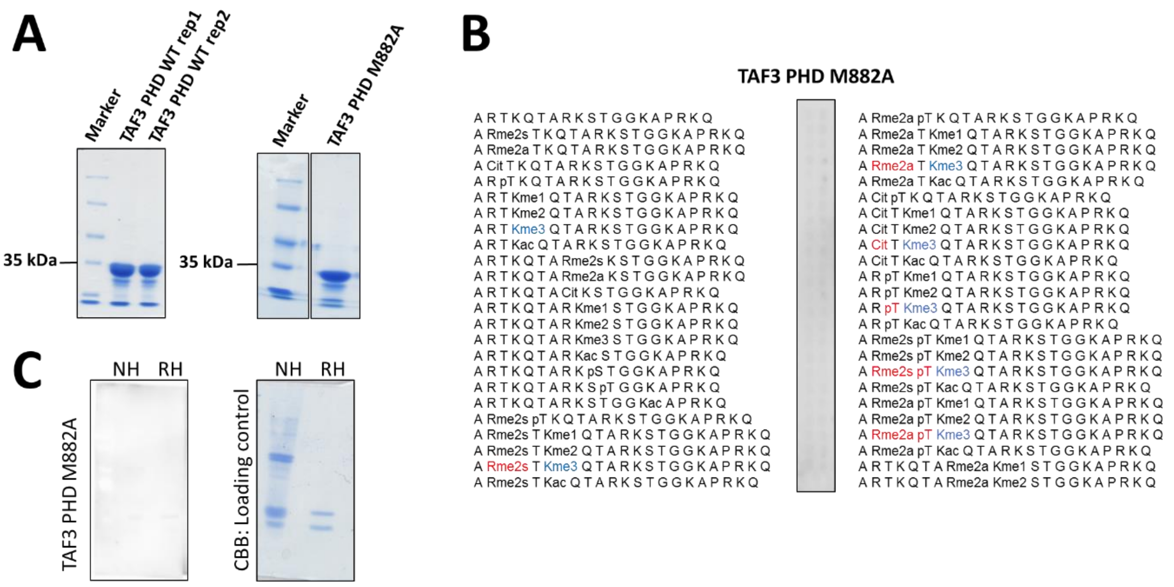

Figure S1. Specificity analyses of TAF3 PHD M882A pocket-mutant. **A)** Coomassie stained SDS-PAGE gel showing the purified GST-fused TAF3 PHD WT and M882A pocket-mutant. **B)** Peptide array profiling of TAF3 PHD M882A does not show binding. **C)** Western blot analyses with TAF3 PHD M882A pocket-mutant with native histones (NH) and recombinant histones (RH) shows loss of binding.

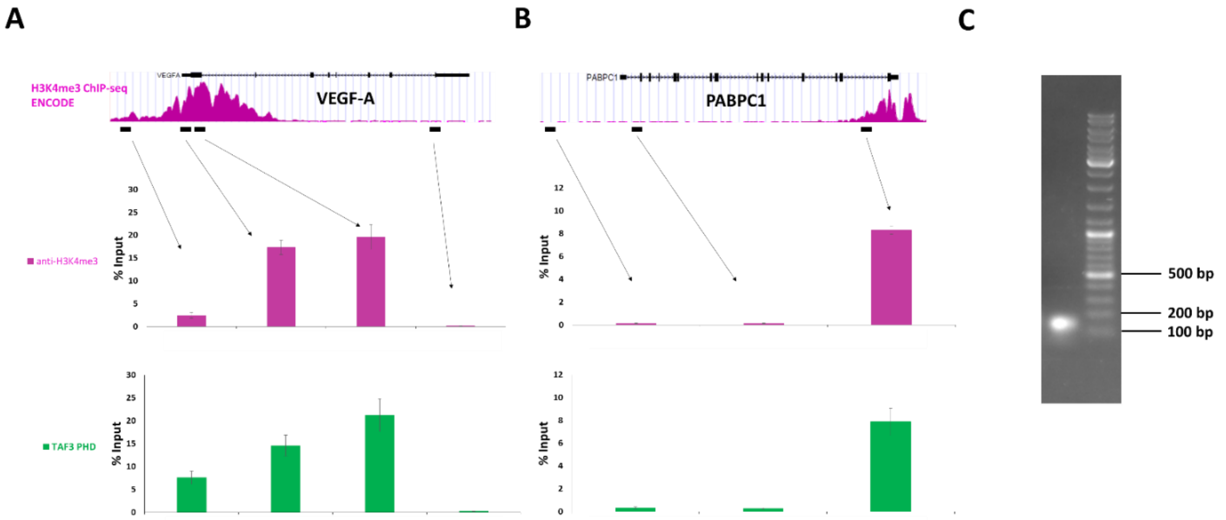

Figure S2. CIDOP and ChIP carried out with TAF3 PHD and anti-H3K4me3 antibody. **A)** CIDOP-qPCR and ChIP-qPCR signal using *VEGF-A* amplicons. **B)** CIDOP-qPCR and ChIP-qPCR signal using *PABPC1* amplicons. The amplicons are annotated as black boxes and the arrows emanating from the amplicons reflect the order of bars in the graph. **C)** Agarose gel electrophoresis of predominantly mononucleosomal DNA used in the CIDOP experiments.

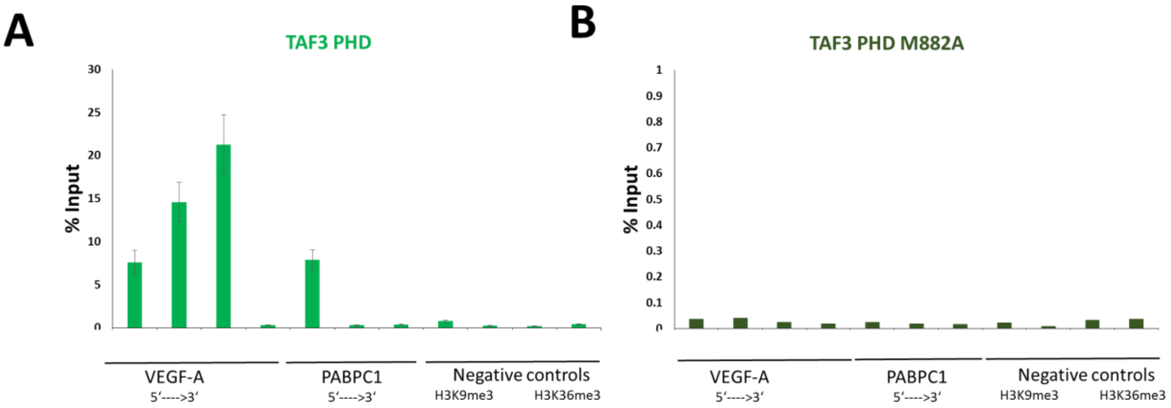

Figure S3. CIDOP-qPCR carried out with TAF3 PHD wild type and TAF3 PHD M882A pocket-mutant. **A)** TAF3 PHD wild type CIDOP-qPCR signals using amplicons covering the *VEGF-A*, *PABPC1* loci and control regions. **B)** TAF3 PHD M882A CIDOP-qPCR signals using amplicons covering the *VEGF-A*, *PABPC1* loci and control regions.

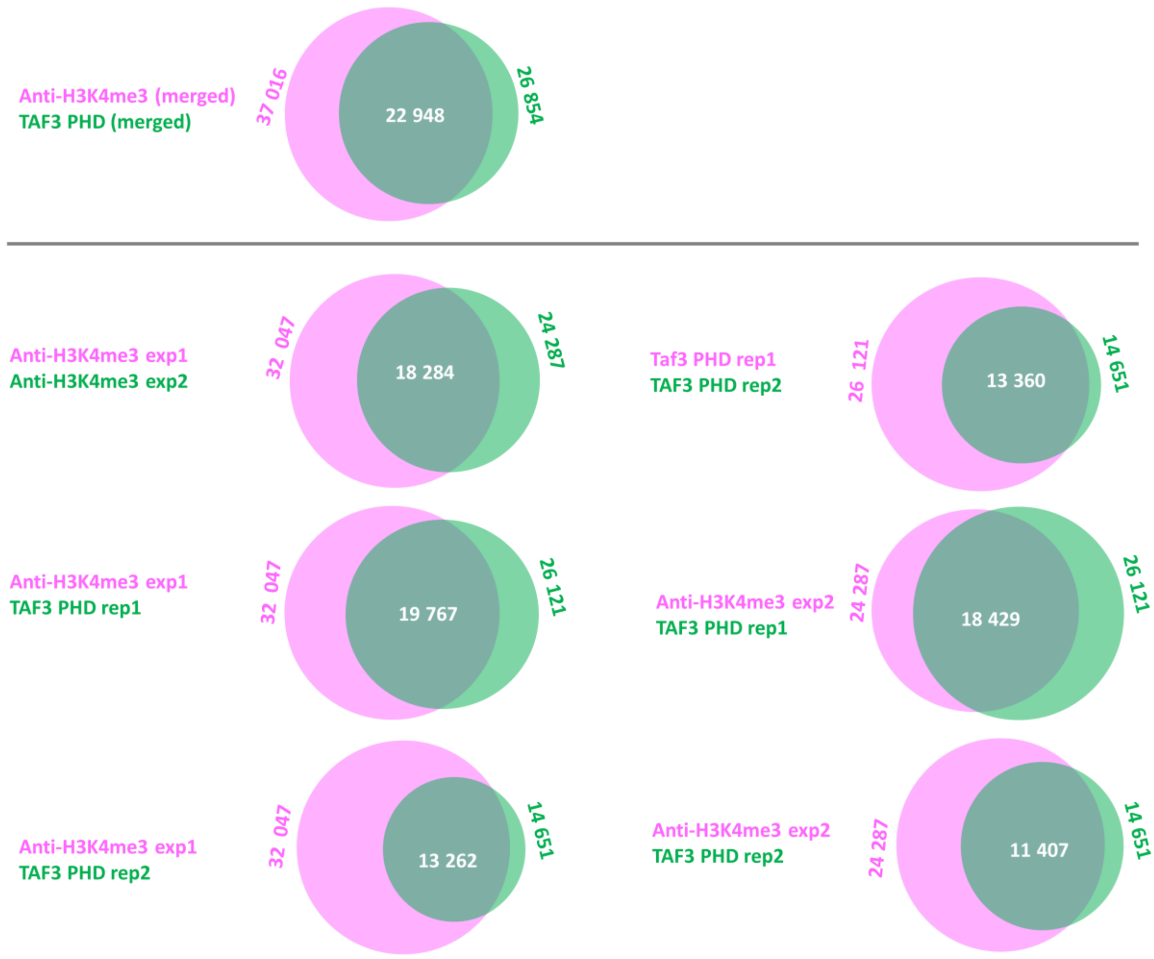

Figure S4. Venn diagrams of the overlap of peak regions between TAF3 PHD and anti-H3K4me3 antibody (merged from both replicates/experiments) and comparison of the individual TAF3 PHD CIDOP-seq and anti-H3K4me3 ChIP-seq replicates/experiments.

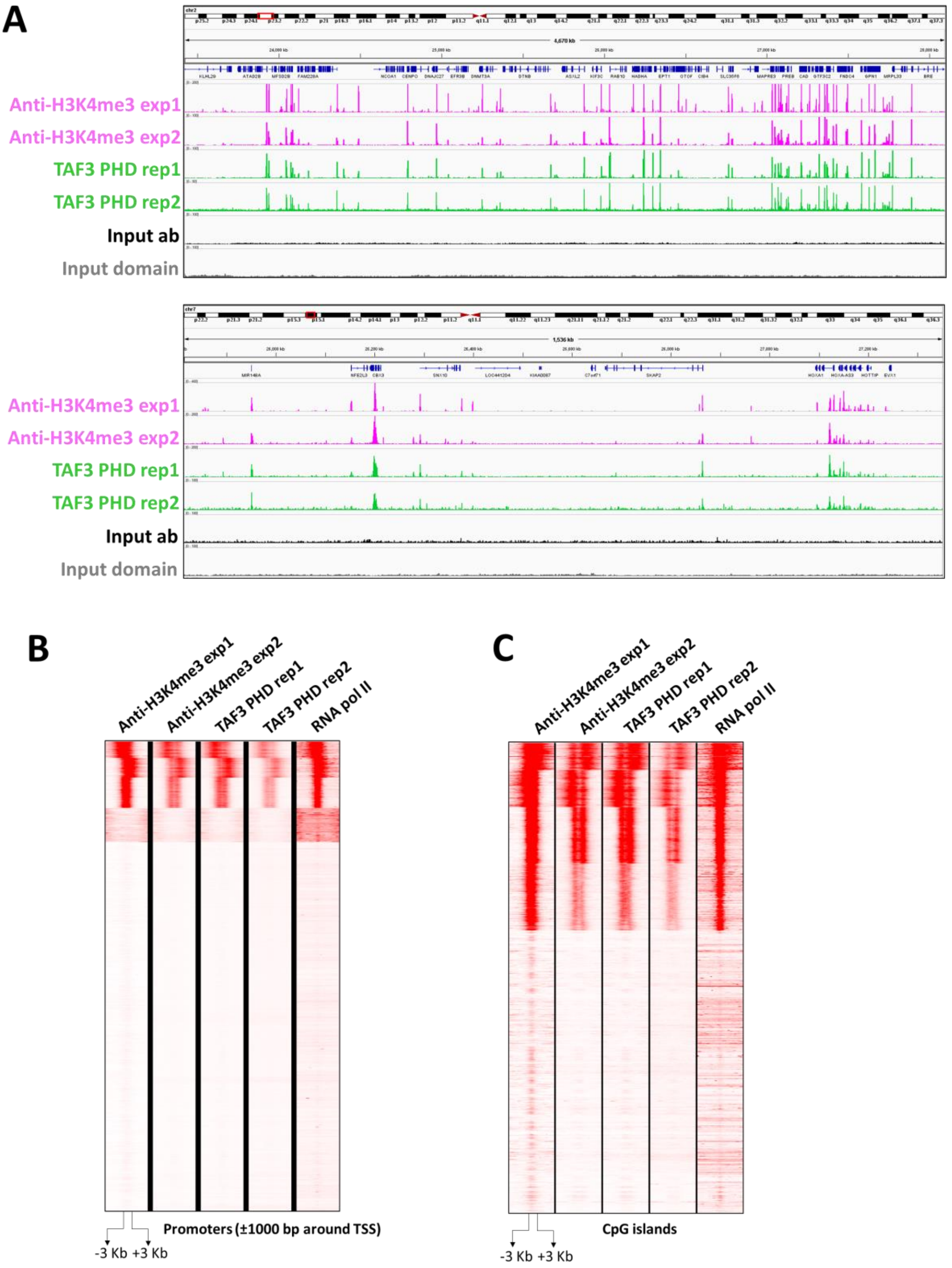

Figure S5. CIDOP-seq and ChIP-seq carried out with TAF3 PHD and anti-H3K4me3 antibody. A) Representative genome browser views comparing CIDOP-seq and ChIP-seq (from ENCODE) signals taken from both replicates/experiments, respectively for each method. B) Clustering analysis of tag densities from TAF3 PHD and anti-H3K4me3 datasets. Tags were collected in 6-kb windows, centered on the midpoints of promoters, and sorted by k-means clustering (5 clusters). C) Clustering analysis of tag densities from TAF3 PHD and anti-H3K4me3 datasets. Tags were collected in 6-kb windows, centered on the midpoints of CpG islands, and sorted by k-means clustering (5 clusters).

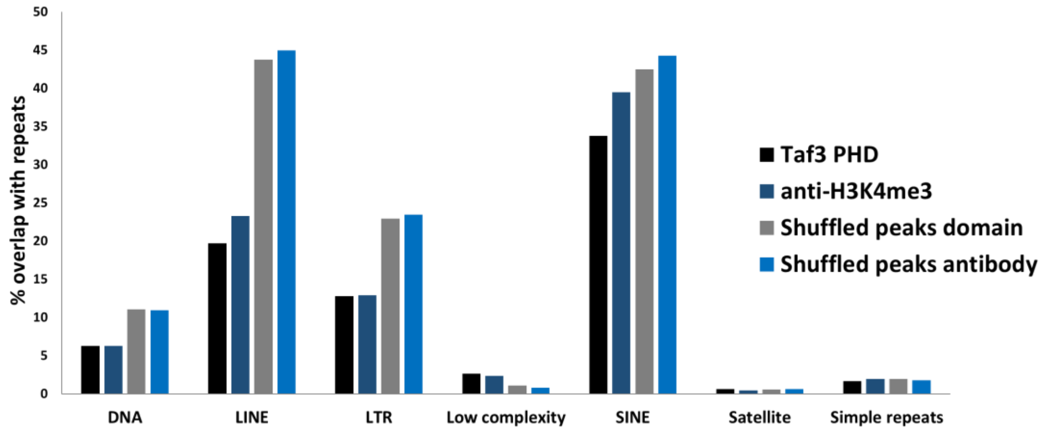

Figure S6. Percent of TAF3 PHD and anti-H3K4me3 peaks (merged from both replicates/experiments, respectively) and randomized shuffled genomic coordinates of the same number showing at least 10% overlap with repeats. Please note the depletion or lack of enrichment in particular with DNA transposons, LINE and LTR elements.

Supplementary tables

Table S1. Primer sequences used in this study.

VEGF-A 5’-3’	Sequences
1	FP 5‘-GCAGTCACTAGGGGGCGCTC-3’<br>RP 5‘-CAACGCCCTCAACCCCACA-3’
2	FP 5‘-ACAGGGGCAAAGTGAGTGAC-3’<br>RP 5‘-GCGGTGTCTGTCTGTCTGTC-3’
3	FP 5‘-CCCCCTCTGTCGTCTTAGGT-3’<br>RP 5‘-AGATCGTACGTGCGGTGACT-3’
4	FP 5‘-GCCCTAACCCCAGCCTTTGTTT-3’<br>RP 5‘-GTATCGATCGTTCTGTATCAGTCTTTCC-3’
PABPC1	
1	FP 5‘-TGACACAGAGCCAGAAGTTGTAAAA -3’<br>RP 5‘-CATGCATTGATACATCCTGCCTAA-3’
2	FP 5‘-CATCACTCCCAAGAAATTGAGTAGT-3’<br>RP 5‘-CACAGTCCTCAAAGCCCAAA-3’
3	
H3K9me3	
1	FP 5‘-ATC GAA TGG AAA TGA AAG GAG TCA-3’<br>RP 5’-5’-GAC CAT TGG ATG ATT GCA GTC A-3’
2	FP 5‘-AGAACACCATGGACCACCAG-3’<br>RP 5‘-TTTCTGAATTGGTTCTGGGTTT-3’
H3K36me3	
1	5‘-FP CTGCTCCCATGTCTGCTACA-3’<br>5‘-RP TGGAAGGACTGCAGAGAAAAA-3’
2	5‘-FP TGCTCCTTTTTCCCATCTTTT-3’<br>5‘-RP GCAAAACCAAGTCGAATGCT-3’
